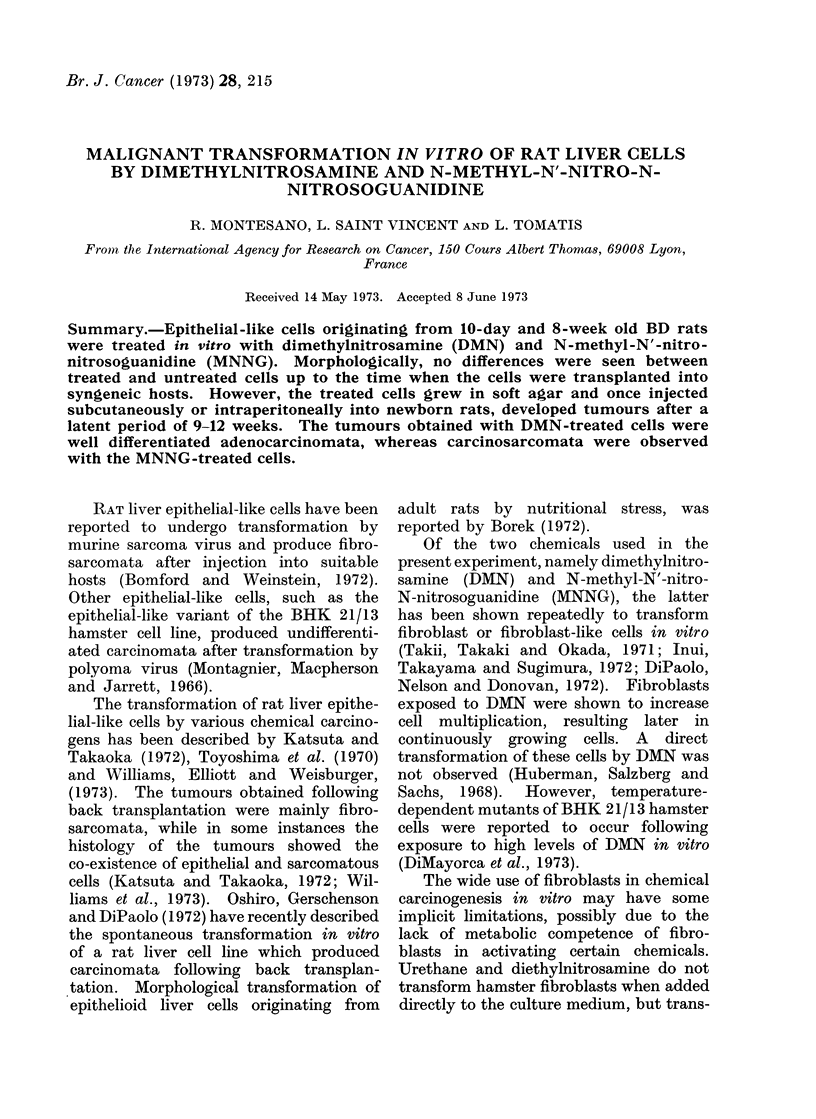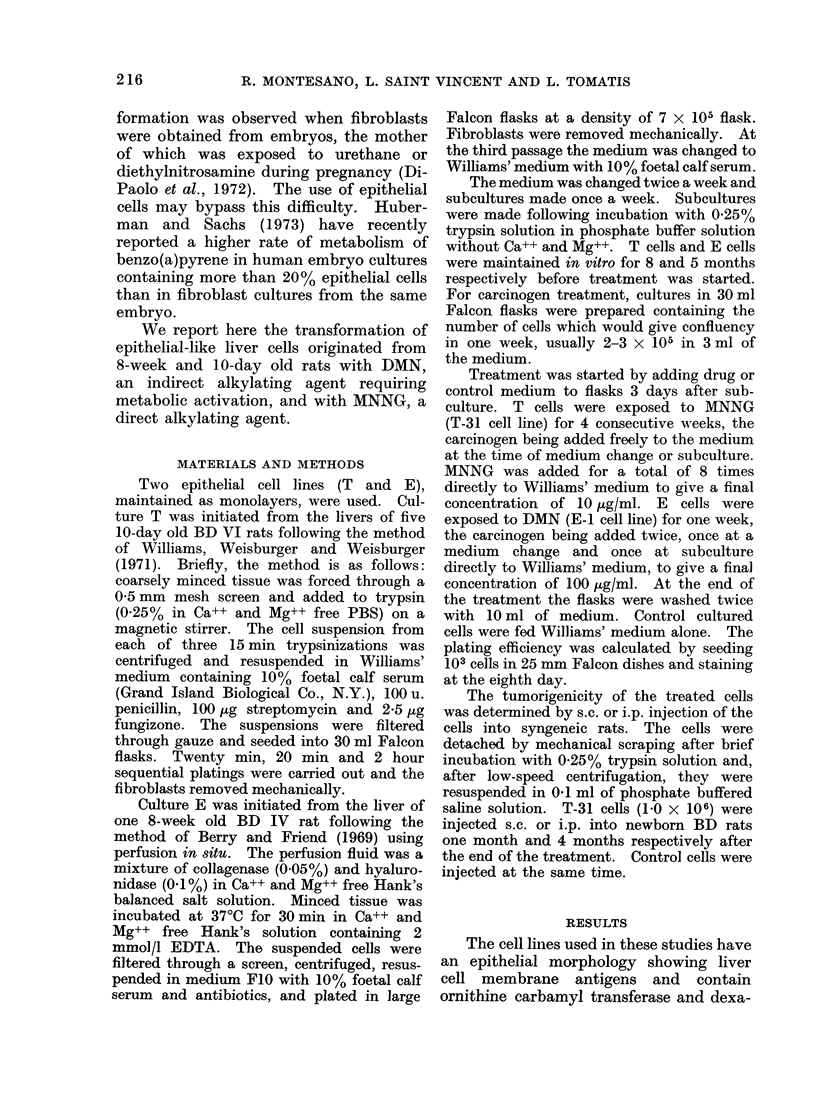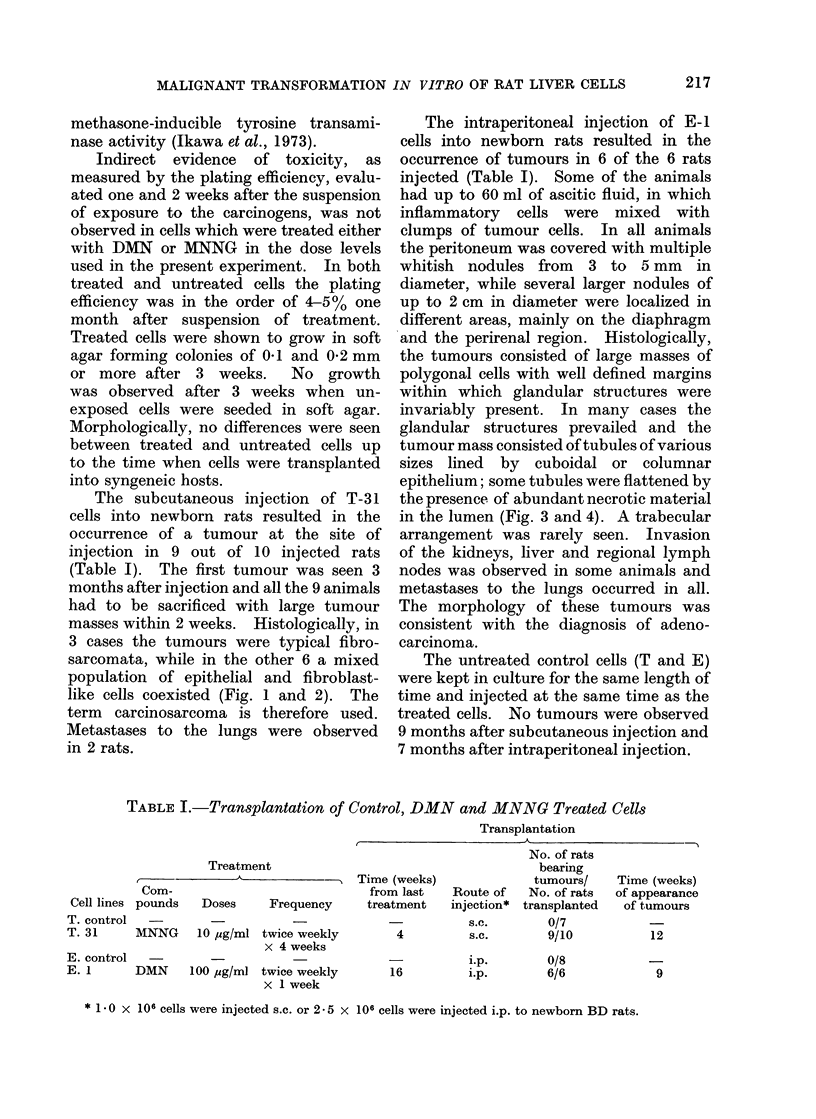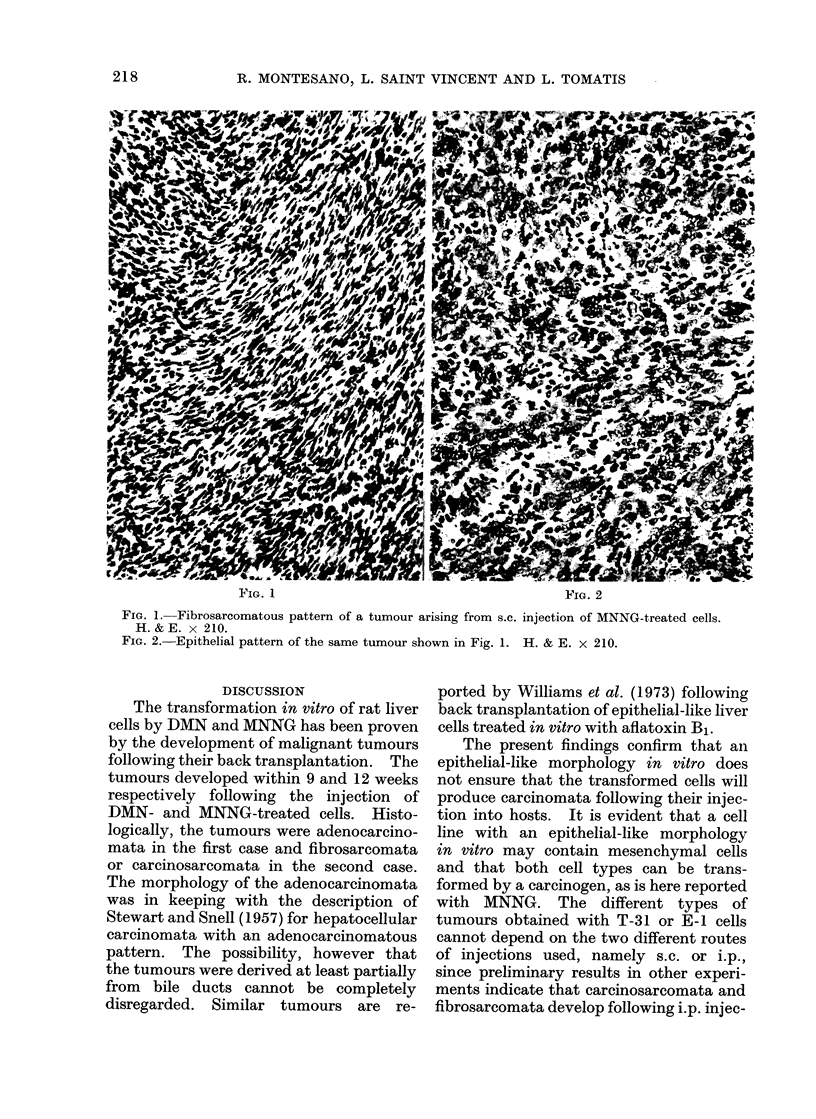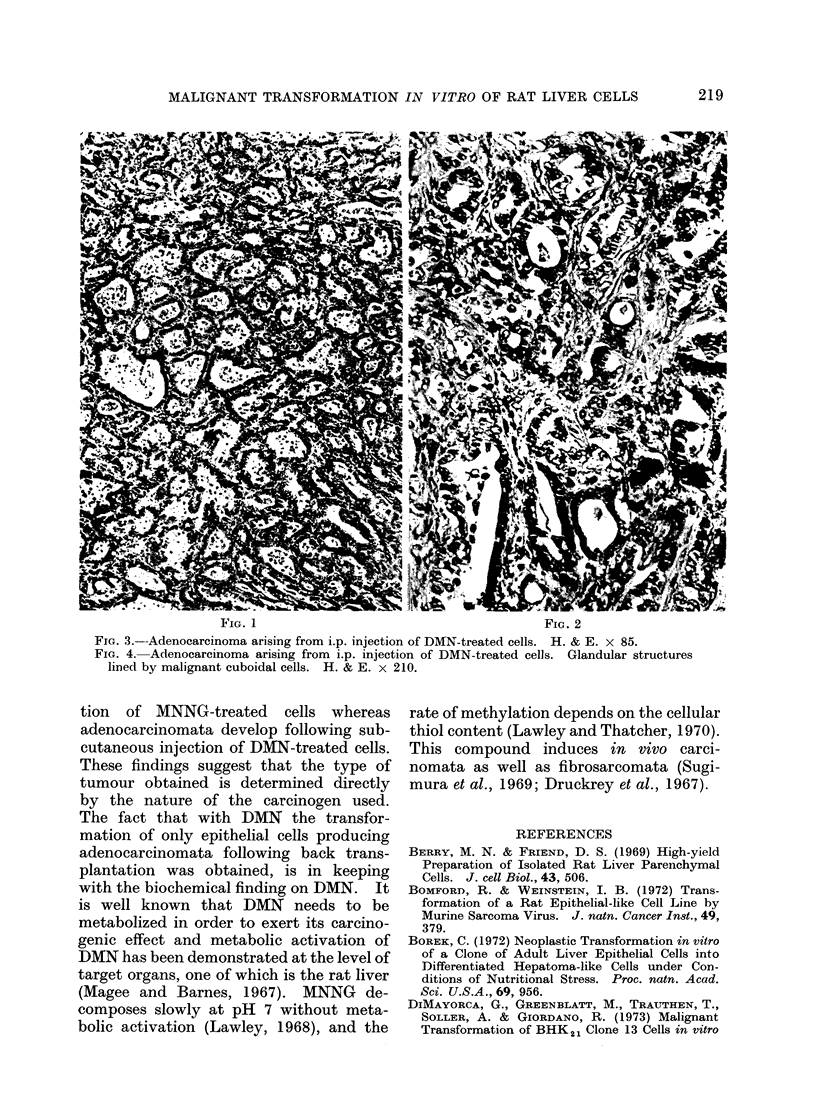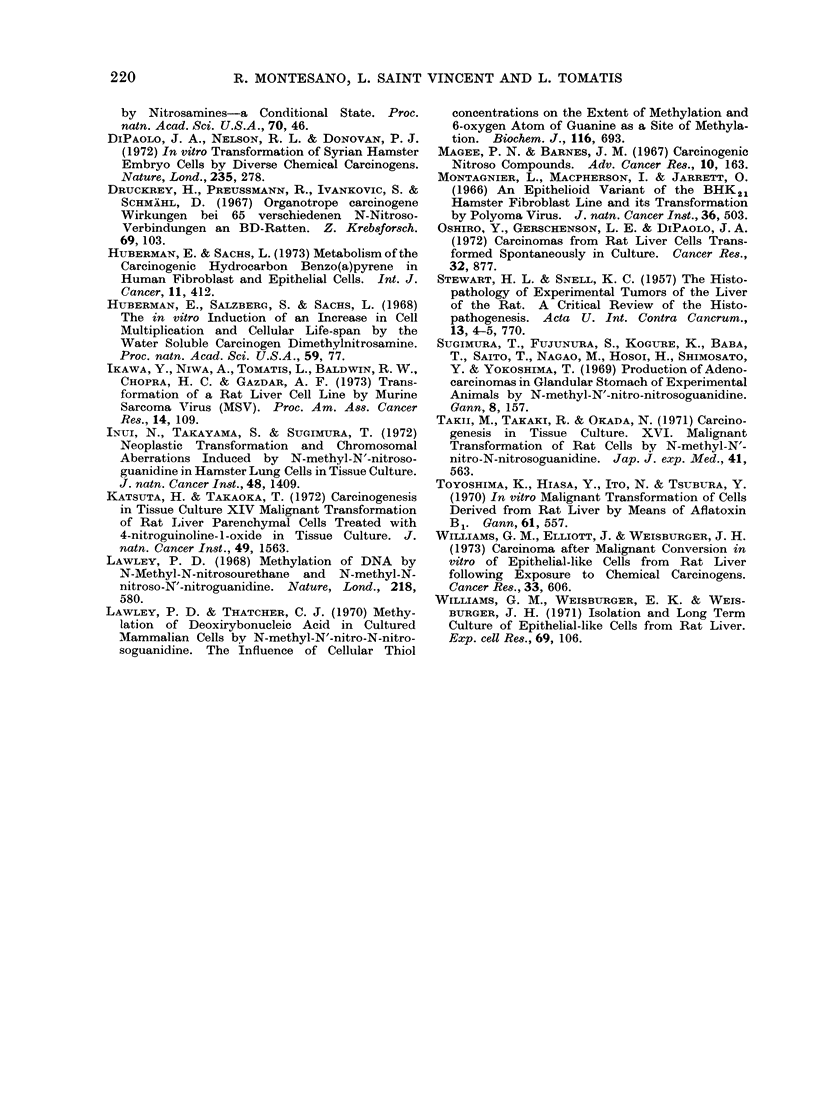# Malignant Transformation in vitro of Rat Liver Cells by Dimethylnitrosamine and N-Methyl-N′-nitro-N-Nitrosoguanidine

**DOI:** 10.1038/bjc.1973.139

**Published:** 1973-09

**Authors:** R. Montesano, L. Saint Vincent, L. Tomatis

## Abstract

**Images:**


*Br. J. Cancer* (1973) **28**, 215

### MALIGNANT TRANSFORMATION *IN VITRO* OF RAT LIVER CELLS BY DIMETHYLNITROSAMINE AND N-METHYL-N′-NITRO-N-NITROSOGUANIDINE

R. MONTESANO, L. SAINT VINCENT AND L. TOMATIS

*From the International Agency for Research on Cancer, 150 Cours Albert Thomas, 69008 Lyon, France*

Received 14 May 1973. Accepted 8 June 1973

**Summary.—Epithelial-like cells originating from 10-day and 8-week old BD rats were treated *in vitro* with dimethylnitrosamine (DMN) and N-methyl-N′-nitro-nitrosoguanidine (MNNG). Morphologically, no differences were seen between treated and untreated cells up to the time when the cells were transplanted into syngeneic hosts. However, the treated cells grew in soft agar and once injected subcutaneously or intraperitoneally into newborn rats, developed tumours after a latent period of 9–12 weeks. The tumours obtained with DMN-treated cells were well differentiated adenocarcinomata, whereas carcinosarcomata were observed with the MNNG-treated cells.**

RAT liver epithelial-like cells have been reported to undergo transformation by murine sarcoma virus and produce fibrosarcomata after injection into suitable hosts (Bomford and Weinstein, 1972). Other epithelial-like cells, such as the epithelial-like variant of the BHK 21/13 hamster cell line, produced undifferentiated carcinomata after transformation by polyoma virus (Montagnier, Macpherson and Jarrett, 1966).

The transformation of rat liver epithelial-like cells by various chemical carcinogens has been described by Katsuta and Takaoka (1972), Toyoshima *et al.* (1970) and Williams, Elliott and Weisburger, (1973). The tumours obtained following back transplantation were mainly fibrosarcomata, while in some instances the histology of the tumours showed the co-existence of epithelial and sarcomatous cells (Katsuta and Takaoka, 1972; Williams *et al.*, 1973). Oshiro, Gerschenson and DiPaolo (1972) have recently described the spontaneous transformation *in vitro* of a rat liver cell line which produced carcinomata following back transplantation. Morphological transformation of epithelioid liver cells originating from adult rats by nutritional stress, was reported by Borek (1972).

Of the two chemicals used in the present experiment, namely dimethylnitrosamine (DMN) and N-methyl-N′-nitro-N-nitrosoguanidine (MNNG), the latter has been shown repeatedly to transform fibroblast or fibroblast-like cells *in vitro* (Takii, Takaki and Okada, 1971; Inui, Takayama and Sugimura, 1972; DiPaolo, Nelson and Donovan, 1972). Fibroblasts exposed to DMN were shown to increase cell multiplication, resulting later in continuously growing cells. A direct transformation of these cells by DMN was not observed (Huberman, Salzberg and Sachs, 1968). However, temperature-dependent mutants of BHK 21/13 hamster cells were reported to occur following exposure to high levels of DMN *in vitro* (DiMayorca *et al.*, 1973).

The wide use of fibroblasts in chemical carcinogenesis *in vitro* may have some implicit limitations, possibly due to the lack of metabolic competence of fibroblasts in activating certain chemicals. Urethane and diethylnitrosamine do not transform hamster fibroblasts when added directly to the culture medium, but transformation was observed when fibroblasts were obtained from embryos, the mother of which was exposed to urethane or diethylnitrosamine during pregnancy (DiPaolo *et al.*, 1972). The use of epithelial cells may bypass this difficulty. Huberman and Sachs (1973) have recently reported a higher rate of metabolism of benzo(a)pyrene in human embryo cultures containing more than 20% epithelial cells than in fibroblast cultures from the same embryo.

We report here the transformation of epithelial-like liver cells originated from 8-week and 10-day old rats with DMN, an indirect alkylating agent requiring metabolic activation, and with MNNG, a direct alkylating agent.

### MATERIALS AND METHODS

Two epithelial cell lines (T and E), maintained as monolayers, were used. Culture T was initiated from the livers of five 10-day old BD VI rats following the method of Williams, Weisburger and Weisburger (1971). Briefly, the method is as follows: coarsely minced tissue was forced through a 0·5 mm mesh screen and added to trypsin (0·25% in $Ca^{++}$ and $Mg^{++}$ free PBS) on a magnetic stirrer. The cell suspension from each of three 15 min trypsinizations was centrifuged and resuspended in Williams' medium containing 10% foetal calf serum (Grand Island Biological Co., N.Y.), 100 u. penicillin, 100 $\mu$g streptomycin and 2·5 $\mu$g fungizone. The suspensions were filtered through gauze and seeded into 30 ml Falcon flasks. Twenty min, 20 min and 2 hour sequential platings were carried out and the fibroblasts removed mechanically.

Culture E was initiated from the liver of one 8-week old BD IV rat following the method of Berry and Friend (1969) using perfusion *in situ*. The perfusion fluid was a mixture of collagenase (0·05%) and hyaluronidase (0·1%) in $Ca^{++}$ and $Mg^{++}$ free Hank's balanced salt solution. Minced tissue was incubated at 37°C for 30 min in $Ca^{++}$ and $Mg^{++}$ free Hank's solution containing 2 mmol/l EDTA. The suspended cells were filtered through a screen, centrifuged, resuspended in medium F10 with 10% foetal calf serum and antibiotics, and plated in large Falcon flasks at a density of $7 \times 10^5$ flask. Fibroblasts were removed mechanically. At the third passage the medium was changed to Williams' medium with 10% foetal calf serum.

The medium was changed twice a week and subcultures made once a week. Subcultures were made following incubation with 0·25% trypsin solution in phosphate buffer solution without $Ca^{++}$ and $Mg^{++}$. T cells and E cells were maintained *in vitro* for 8 and 5 months respectively before treatment was started. For carcinogen treatment, cultures in 30 ml Falcon flasks were prepared containing the number of cells which would give confluency in one week, usually $2–3 \times 10^5$ in 3 ml of the medium.

Treatment was started by adding drug or control medium to flasks 3 days after subculture. T cells were exposed to MNNG (T-31 cell line) for 4 consecutive weeks, the carcinogen being added freely to the medium at the time of medium change or subculture. MNNG was added for a total of 8 times directly to Williams' medium to give a final concentration of 10 $\mu$g/ml. E cells were exposed to DMN (E-1 cell line) for one week, the carcinogen being added twice, once at a medium change and once at subculture directly to Williams' medium, to give a final concentration of 100 $\mu$g/ml. At the end of the treatment the flasks were washed twice with 10 ml of medium. Control cultured cells were fed Williams' medium alone. The plating efficiency was calculated by seeding $10^3$ cells in 25 mm Falcon dishes and staining at the eighth day.

The tumorigenicity of the treated cells was determined by s.c. or i.p. injection of the cells into syngeneic rats. The cells were detached by mechanical scraping after brief incubation with 0·25% trypsin solution and, after low-speed centrifugation, they were resuspended in 0·1 ml of phosphate buffered saline solution. T-31 cells ($1·0 \times 10^6$) were injected s.c. or i.p. into newborn BD rats one month and 4 months respectively after the end of the treatment. Control cells were injected at the same time.

### RESULTS

The cell lines used in these studies have an epithelial morphology showing liver cell membrane antigens and contain ornithine carbamyl transferase and dexamethasone-inducible tyrosine transaminase activity (Ikawa *et al.*, 1973).

Indirect evidence of toxicity, as measured by the plating efficiency, evaluated one and 2 weeks after the suspension of exposure to the carcinogens, was not observed in cells which were treated either with DMN or MNNG in the dose levels used in the present experiment. In both treated and untreated cells the plating efficiency was in the order of 4–5% one month after suspension of treatment. Treated cells were shown to grow in soft agar forming colonies of 0·1 and 0·2 mm or more after 3 weeks. No growth was observed after 3 weeks when unexposed cells were seeded in soft agar. Morphologically, no differences were seen between treated and untreated cells up to the time when cells were transplanted into syngeneic hosts.

The subcutaneous injection of T-31 cells into newborn rats resulted in the occurrence of a tumour at the site of injection in 9 out of 10 injected rats (Table I). The first tumour was seen 3 months after injection and all the 9 animals had to be sacrificed with large tumour masses within 2 weeks. Histologically, in 3 cases the tumours were typical fibrosarcomata, while in the other 6 a mixed population of epithelial and fibroblast-like cells coexisted (Fig. 1 and 2). The term carcinosarcoma is therefore used. Metastases to the lungs were observed in 2 rats.

The intraperitoneal injection of E-1 cells into newborn rats resulted in the occurrence of tumours in 6 of the 6 rats injected (Table I). Some of the animals had up to 60 ml of ascitic fluid, in which inflammatory cells were mixed with clumps of tumour cells. In all animals the peritoneum was covered with multiple whitish nodules from 3 to 5 mm in diameter, while several larger nodules of up to 2 cm in diameter were localized in different areas, mainly on the diaphragm and the perirenal region. Histologically, the tumours consisted of large masses of polygonal cells with well defined margins within which glandular structures were invariably present. In many cases the glandular structures prevailed and the tumour mass consisted of tubules of various sizes lined by cuboidal or columnar epithelium; some tubules were flattened by the presence of abundant necrotic material in the lumen (Fig. 3 and 4). A trabecular arrangement was rarely seen. Invasion of the kidneys, liver and regional lymph nodes was observed in some animals and metastases to the lungs occurred in all. The morphology of these tumours was consistent with the diagnosis of adenocarcinoma.

The untreated control cells (T and E) were kept in culture for the same length of time and injected at the same time as the treated cells. No tumours were observed 9 months after subcutaneous injection and 7 months after intraperitoneal injection.

TABLE I.—*Transplantation of Control, DMN and MNNG Treated Cells*

| | Treatment | | | Transplantation | | | |
|---|---|---|---|---|---|---|---|
| Cell lines | Compounds | Doses | Frequency | Time (weeks) from last treatment | Route of injection* | No. of rats bearing tumours/ No. of rats transplanted | Time (weeks) of appearance of tumours |
| T. control | — | — | — | — | s.c. | 0/7 | — |
| T. 31 | MNNG | 10 μg/ml | twice weekly × 4 weeks | 4 | s.c. | 9/10 | 12 |
| E. control | — | — | — | — | i.p. | 0/8 | — |
| E. 1 | DMN | 100 μg/ml | twice weekly × 1 week | 16 | i.p. | 6/6 | 9 |

* $1 \cdot 0 \times 10^6$ cells were injected s.c. or $2 \cdot 5 \times 10^6$ cells were injected i.p. to newborn BD rats.

FIG. 1

FIG. 2

FIG. 1.—Fibrosarcomatous pattern of a tumour arising from s.c. injection of MNNG-treated cells. H. & E. × 210.

FIG. 2.—Epithelial pattern of the same tumour shown in Fig. 1. H. & E. × 210.

### DISCUSSION

The transformation *in vitro* of rat liver cells by DMN and MNNG has been proven by the development of malignant tumours following their back transplantation. The tumours developed within 9 and 12 weeks respectively following the injection of DMN- and MNNG-treated cells. Histologically, the tumours were adenocarcinomata in the first case and fibrosarcomata or carcinosarcomata in the second case. The morphology of the adenocarcinomata was in keeping with the description of Stewart and Snell (1957) for hepatocellular carcinomata with an adenocarcinomatous pattern. The possibility, however that the tumours were derived at least partially from bile ducts cannot be completely disregarded. Similar tumours are reported by Williams *et al.* (1973) following back transplantation of epithelial-like liver cells treated *in vitro* with aflatoxin $B_1$.

The present findings confirm that an epithelial-like morphology *in vitro* does not ensure that the transformed cells will produce carcinomata following their injection into hosts. It is evident that a cell line with an epithelial-like morphology *in vitro* may contain mesenchymal cells and that both cell types can be transformed by a carcinogen, as is here reported with MNNG. The different types of tumours obtained with T-31 or E-1 cells cannot depend on the two different routes of injections used, namely s.c. or i.p., since preliminary results in other experiments indicate that carcinosarcomata and fibrosarcomata develop following i.p. injec-

FIG. 1

FIG. 2

FIG. 3.—Adenocarcinoma arising from i.p. injection of DMN-treated cells. H. & E. × 85.

FIG. 4.—Adenocarcinoma arising from i.p. injection of DMN-treated cells. Glandular structures lined by malignant cuboidal cells. H. & E. × 210.

tion of MNNG-treated cells whereas adenocarcinomata develop following subcutaneous injection of DMN-treated cells. These findings suggest that the type of tumour obtained is determined directly by the nature of the carcinogen used. The fact that with DMN the transformation of only epithelial cells producing adenocarcinomata following back transplantation was obtained, is in keeping with the biochemical finding on DMN. It is well known that DMN needs to be metabolized in order to exert its carcinogenic effect and metabolic activation of DMN has been demonstrated at the level of target organs, one of which is the rat liver (Magee and Barnes, 1967). MNNG decomposes slowly at pH 7 without metabolic activation (Lawley, 1968), and the rate of methylation depends on the cellular thiol content (Lawley and Thatcher, 1970). This compound induces *in vivo* carcinomata as well as fibrosarcomata (Sugimura *et al.*, 1969; Druckrey *et al.*, 1967).

### REFERENCES

BERRY, M. N. & FRIEND, D. S. (1969) High-yield Preparation of Isolated Rat Liver Parenchymal Cells. *J. cell Biol.*, **43**, 506.

BOMFORD, R. & WEINSTEIN, I. B. (1972) Transformation of a Rat Epithelial-like Cell Line by Murine Sarcoma Virus. *J. natn. Cancer Inst.*, **49**, 379.

BOREK, C. (1972) Neoplastic Transformation *in vitro* of a Clone of Adult Liver Epithelial Cells into Differentiated Hepatoma-like Cells under Conditions of Nutritional Stress. *Proc. natn. Acad. Sci. U.S.A.*, **69**, 956.

DIMAYORCA, G., GREENBLATT, M., TRAUTHEN, T., SOLLER, A. & GIORDANO, R. (1973) Malignant Transformation of $BHK_{21}$ Clone 13 Cells *in vitro*

by Nitrosamines—a Conditional State. *Proc. natn. Acad. Sci. U.S.A.*, **70**, 46.

DiPaolo, J. A., Nelson, R. L. & Donovan, P. J. (1972) *In vitro* Transformation of Syrian Hamster Embryo Cells by Diverse Chemical Carcinogens. *Nature, Lond.*, **235**, 278.

Druckrey, H., Preussmann, R., Ivankovic, S. & Schmähl, D. (1967) Organotrope carcinogene Wirkungen bei 65 verschiedenen N-Nitroso-Verbindungen an BD-Ratten. *Z. Krebsforsch.* **69**, 103.

Huberman, E. & Sachs, L. (1973) Metabolism of the Carcinogenic Hydrocarbon Benzo(a)pyrene in Human Fibroblast and Epithelial Cells. *Int. J. Cancer*, **11**, 412.

Huberman, E., Salzberg, S. & Sachs, L. (1968) The *in vitro* Induction of an Increase in Cell Multiplication and Cellular Life-span by the Water Soluble Carcinogen Dimethylnitrosamine. *Proc. natn. Acad. Sci. U.S.A.*, **59**, 77.

Ikawa, Y., Niwa, A., Tomatis, L., Baldwin, R. W., Chopra, H. C. & Gazdar, A. F. (1973) Transformation of a Rat Liver Cell Line by Murine Sarcoma Virus (MSV). *Proc. Am. Ass. Cancer Res.*, **14**, 109.

Inui, N., Takayama, S. & Sugimura, T. (1972) Neoplastic Transformation and Chromosomal Aberrations Induced by N-methyl-N′-nitrosoguanidine in Hamster Lung Cells in Tissue Culture. *J. natn. Cancer Inst.*, **48**, 1409.

Katsuta, H. & Takaoka, T. (1972) Carcinogenesis in Tissue Culture XIV Malignant Transformation of Rat Liver Parenchymal Cells Treated with 4-nitroguinoline-1-oxide in Tissue Culture. *J. natn. Cancer Inst.*, **49**, 1563.

Lawley, P. D. (1968) Methylation of DNA by N-Methyl-N-nitrosourethane and N-methyl-N-nitroso-N′-nitroguanidine. *Nature, Lond.*, **218**, 580.

Lawley, P. D. & Thatcher, C. J. (1970) Methylation of Deoxirybonucleic Acid in Cultured Mammalian Cells by N-methyl-N′-nitro-N-nitrosoguanidine. The Influence of Cellular Thiol concentrations on the Extent of Methylation and 6-oxygen Atom of Guanine as a Site of Methylation. *Biochem. J.*, **116**, 693.

Magee, P. N. & Barnes, J. M. (1967) Carcinogenic Nitroso Compounds. *Adv. Cancer Res.*, **10**, 163.

Montagnier, L., Macpherson, I. & Jarrett, O. (1966) An Epithelioid Variant of the $BHK_{21}$ Hamster Fibroblast Line and its Transformation by Polyoma Virus. *J. natn. Cancer Inst.*, **36**, 503.

Oshiro, Y., Gerschenson, L. E. & DiPaolo, J. A. (1972) Carcinomas from Rat Liver Cells Transformed Spontaneously in Culture. *Cancer Res.*, **32**, 877.

Stewart, H. L. & Snell, K. C. (1957) The Histopathology of Experimental Tumors of the Liver of the Rat. A Critical Review of the Histopathogenesis. *Acta U. Int. Contra Cancrum.*, **13**, 4–5, 770.

Sugimura, T., Fujunura, S., Kogure, K., Baba, T., Saito, T., Nagao, M., Hosoi, H., Shimosato, Y. & Yokoshima, T. (1969) Production of Adenocarcinomas in Glandular Stomach of Experimental Animals by N-methyl-N′-nitro-nitrosoguanidine. *Gann*, **8**, 157.

Takii, M., Takaki, R. & Okada, N. (1971) Carcinogenesis in Tissue Culture. XVI. Malignant Transformation of Rat Cells by N-methyl-N′-nitro-N-nitrosoguanidine. *Jap. J. exp. Med.*, **41**, 563.

Toyoshima, K., Hiasa, Y., Ito, N. & Tsubura, Y. (1970) *In vitro* Malignant Transformation of Cells Derived from Rat Liver by Means of Aflatoxin $B_1$. *Gann*, **61**, 557.

Williams, G. M., Elliott, J. & Weisburger, J. H. (1973) Carcinoma after Malignant Conversion *in vitro* of Epithelial-like Cells from Rat Liver following Exposure to Chemical Carcinogens. *Cancer Res.*, **33**, 606.

Williams, G. M., Weisburger, E. K. & Weisburger, J. H. (1971) Isolation and Long Term Culture of Epithelial-like Cells from Rat Liver. *Exp. cell Res.*, **69**, 106.